# Assessment of the tumourigenic and metastatic properties of SK-MEL28 melanoma cells surviving electrochemotherapy with bleomycin

**DOI:** 10.2478/v10019-012-0010-6

**Published:** 2012-01-12

**Authors:** Vesna Todorovic, Gregor Sersa, Vid Mlakar, Damjan Glavac, Maja Cemazar

**Affiliations:** 1 Institute of Oncology Ljubljana, Department of Experimental Oncology, Ljubljana, Slovenia; 2 University of Primorska, Faculty of Health Sciences, Izola, Slovenia; 3 University of Ljubljana, Faculty of Medicine, Institute of Pathology, Department of Molecular Genetics, Ljubljana, Slovenia

**Keywords:** bleomycin, electrochemotherapy, electroporation, metastatic potential, melanoma, microarray analysis

## Abstract

**Background:**

Electrochemotherapy is a local treatment combining chemotherapy and electroporation and is highly effective treatment approach for subcutaneous tumours of various histologies. Contrary to surgery and radiation, the effect of electrochemotherapy on metastatic potential of tumour cells has not been extensively studied. The aim of the study was to evaluate the effect of electrochemotherapy with bleomycin on the metastatic potential of human melanoma cells *in vitro*.

**Materials and methods:**

Viable cells 48 hours after electrochemotherapy were tested for their ability to migrate and invade through Matrigel coated porous membrane. In addition, microarray analysis and quantitative Real-Time PCR were used to detect changes in gene expression after electrochemotherapy.

**Results:**

Cell migration and invasion were not changed in melanoma cells surviving electrochemotherapy. Interestingly, only a low number of tumourigenesis related genes was differentially expressed after electrochemotherapy.

**Conclusions:**

Our data suggest that metastatic potential of human melanoma cells is not affected by electrochemotherapy with bleomycin, confirming safe role of electrochemotherapy in the clinics.

## Introduction

Metastatic progression is a complex multi-step process that requires acquisition of many specific cell properties, such as loss of cellular adhesion, increased invasiveness, intravasation and survival in the vascular system, extravasation, survival and proliferation in a new microenvironment. Each of these properties is fulfilling a specific function in the metastatic cascade for successful establishment of metastases.^1^,^2^ Interactions between metastatic cells and their microenvironment are important for the development of metastasis.^1^ Random genetic and epigenetic alterations in cancer cells in a combination with a plastic and responsive microenvironment support the metastatic evolution of tumours.^3^

It is now evident that alterations in tumour cells and/or their microenvironment induced by therapy can affect interactions between metastatic cells and their microenvironment, and thus play an important role in metastasis induction. Experimental studies demonstrated that surgical resection of a tumour changes the microenvironment of the wound site and provides a microenvironment favourable for tumour growth.^4^–^6^ Significantly fewer malignant cells are required to grow a tumour in the wounded tissue.^6^ Depending on the type of cancer, tumour growth in the wounded tissue is accelerated at different degree.^4^ In addition, studies of experimental tumours have confirmed radiation-induced metastases.^7^ Depending on the type and dose of radiation used, a decrease or increase in cell migration and invasion can be observed in different tumour types *in vitro* and *in vivo*.^7^–^11^

Electrochemotherapy (ECT) is a local treatment combining chemotherapy and electroporation. Electroporation is a highly effective physical method for transient modification of cell membrane permeability by means of series of controlled electric pulses.^12^,^13^ Different studies demonstrated that electroporation is effective in facilitating uptake of different molecules into cells, including chemotherapeutic drugs.^14^ Among these, chemotherapeutic drugs bleomycin (BLM) and cisplatin proved to be effective in ECT. Electroporation increased BLM cytotoxicity several thousand fold, and cisplatin cytotoxicity up to 80-fold.^15^ Clinical trials demonstrated that ECT with BLM or cisplatin is effective in the treatment of skin tumour nodules of various tumours, including malignant melanoma.^15^–^19^

In contrast to surgery and radiation, the effect of ECT on metastatic potential of tumour cells has only recently gained attention. Although there were no reports of increased metastatic spread of tumours after ECT in the clinical studies, it is important to evaluate the effect of suboptimal therapy on tumour cells.^20^ Namely, biological properties of tumour cells that were suboptimally affected by ECT due to insufficient drug distribution or suboptimal electroporation of the tissue can be modified and thus represent a relevant problem.^21^,^22^ Till date, it was shown that ECT with BLM does not increase metastatic spread of liver tumours in rabbits.^23^ In clinical trials, there were no reports of increased metastatic spread of tumours after ECT.^20^ None of the lesions in complete response after ECT with BLM relapsed during the follow-up of 21 months.^18^ Previously, it was demonstrated that electroporation alone does not significantly change the expression of major cancer related genes.^24^ Also, metastatic potential of melanoma cells *in vitro* is not affected by ECT with cisplatin.^25^ The results of both studies are supporting current evidence that electroporation and ECT are safe methods that do not induce tumour progression. However, the effect of electrochemotherapy with BLM, the most used drug in electrochemotherapy, on the metastatic potential is not known.

Therefore, the aim of the current study was to assess for the first time the effect of ECT with BLM on the metastatic potential of melanoma cells *in vitro*. Furthermore, we also evaluated its effect on gene expression in the same cells. For this purpose, cell migration and invasion of cells surviving ECT with BLM were evaluated using porous cell culture inserts widely used in *in vitro* studies of cell migration and invasion.^26^,^27^ Furthermore, the effect of ECT with BLM on gene expression was determined by microarrays and validation of gene expression of differentially expressed genes involved in metastatic process was performed by qRT-PCR.

## Materials and methods

### Cell line

Human malignant melanoma cells SK-MEL28 (HTB-72; American Type Culture Collection, USA) were derived from a melanoma metastasis and have high migratory and moderate invasive potential.^28^,^29^ SK-MEL28 were grown as monolayer in Minimum Essential Medium (MEM) with Glutamax (Gibco, Invitrogen, Paisley, UK), supplemented with 10% foetal calf serum (FCS) (Invitrogen, Paisley, UK) and gentamicin (30 μg/ mL) (Gibco, Invitrogen, Paisley, UK). Cells were routinely subcultured twice a week and incubated in an atmosphere with 5% CO_2_ at 37°C.

### Drug

BLM (Blenamax) was obtained from Pharmachemie BV (Haarlem, the Netherlands) as a crystalline powder. BLM was dissolved in saline (0.9% NaCl) at a concentration 1 mM. For each experiment, a fresh solution of BLM was prepared. The final concentrations of BLM (0.01 nM to 1 μM) were prepared in DMEM.

### Electrochemotherapy protocol

Confluent cell cultures were trypsinized, washed in MEM with FCS for trypsin inactivation and once in electroporation buffer (125 mM sucrose; 10 mM K_2_HPO_4_; 2.5 mM KH_2_PO_4_; 2 mM MgCl_2_·6H_2_0) at 4°C. The final cell suspension was prepared in electroporation buffer at 4°C at a concentration of 22 × 10^6^ cells/mL. For clonogenic assay, 90 μL (2 × 10^6^ cells) of the final cell suspension was mixed with 10 μL of BLM solution in concentration range 0.00001 μM to 1 μM. For microarray assay, 270 μL (6 × 10^6^ cells) of the final cell suspension was mixed with 10 μL of 0.1 μM BLM. One half of the mixture served as a control of BLM treatment alone. The other half of the mixture was placed between two parallel electrodes with 2 mm gap in between and subjected to eight square wave electric pulses with electric field intensity 1300 V/cm, pulse duration 100 μs and frequency 1 Hz. Electric pulses were generated by in-house build electroporator (University of Ljubljana, Faculty of Electrical Engineering, Ljubljana, Slovenia). After electroporation cells were incubated at room temperature for 5 minutes, diluted in 2 mL of growth media and then plated for clonogenic, microarray and qRT-PCR assays.

### Cell survival and viability after electrochemotherapy

Clonogenic assay was used to determine cell survival after exposure to BLM, electroporation and ECT. After exposure to BLM alone, electroporation, or ECT with BLM, SK-MEL28 were plated at a concentration of 300 to 1200 cells/dish. After 15 days, colonies were fixed, stained with crystal violet and counted. The plating efficiency and the surviving fraction were calculated. The surviving fraction of cells exposed to electrochemotherapy was normalized to electric pulses treatment alone. The experiments were performed in triplicate and repeated three times.

Cell viability assay (MTS assay, Promega, Madison, USA) was used to determine cell proliferation 48 and 72 h after ECT. After ECT protocol, 1.5 × 10^4^ cells/well were seeded in two separate 96 well plates and left for 48 h and 72 h. After 48 h and 72 h, a solution of MTS with PMS (ratio 20:1) was added to each well and after 2 h absorbance was measured at 492 nm using microplate reader (Tecan, Salzburg, Austria). Absorbance at 492 nm is directly proportional to cell viability and was normalized to control cells at 48 h for each sample. The experiment was repeated twice in sextuplicates.

### Migration and invasion assay

For migration assay, uncoated inserts with polycarbonate membrane with 8 μm pores (TPP, Switzerland) in 24 well plates were used. 48 h after exposure to BLM or ECT, cells were trypsinized, washed in MEM with FCS for trypsin inactivation, centrifuged, resuspended in serum-free MEM and counted. Cells (9 × 10^4^ per well) were plated in inserts (TPP) with 8 μm pores in 24 well plate in 400 μL serum-free MEM and 400 μL MEM with 10% FCS as chemo-attractant was added to the wells in 24 well plate. After 22 h, MTT was added to inserts and wells and incubated for another 2 h. The migrated cells were washed off the bottom of the insert and collected in the original well, whereas the inserts were transferred to a clean well. Formazan crystals were dissolved in dimethyl sulfoxide (DMSO). Absorbance was measured at 595 nm using microplate reader (Tecan). The experiment was repeated three times in quadruplicates. Migration is the ratio between the absorbance of the cells collected in the original well over the sum of absorbance of the cells collected in the original well and the cells collected in the insert.

For invasion assay, the inside of the insert was coated with Matrigel (BD Bioscience, USA) diluted in serum-free MEM according to manufacturer’s instructions. Inserts were incubated for 1 h at room temperature to allow Matrigel polymerization. The inserts were washed with serum-free MEM. Invasion was tested in the presence of Matrigel but otherwise as described for the migration assay. The experiment was repeated three times in quadruplicates. Invasion is the ratio between absorbance of the cells collected in the original well over the sum of absorbance of the cells collected in the original well and the cells collected in the insert.

### Adhesion assay

Cell adhesion assay was determined 48 h post-treatment. For adhesion assay, 96 well plate was coated with Matrigel diluted in serum-free MEM according to manufacturer’s instructions. 96 well plates were incubated for 1 h at room temperature to allow Matrigel polymerization. Unbound material was aspirated and 96 well plates were gently washed with serum-free MEM. BLM or ECT treated cells were trypsinized, washed in MEM with serum for trypsin inactivation, centrifuged, resuspended in serum-free MEM and counted. Cells (3 × 10^4^ per well) were plated in pre-prepared 96 well plate in 200 μL serum-free MEM. After 2 h, medium and unbound cells were removed from the wells and attached cells were gently washed with 1x PBS and fresh serum-free MEM with MTT (0.5 mg/mL) (Calbiochem, Germany) was added. Cells were incubated for another 2 h, media was then removed and formazan crystals dissolved in DMSO (Sigma Aldrich, Steinheim, Germany). Absorbance was measured at 595 nm using microplate reader (Tecan). The experiment was repeated four times in septuplicates.

### Microarray assay

RNA from BLM and ECT treated cells (with 0.1 μM) was isolated using TRI REAGENT^™^ (Sigma Aldrich, Germany) and a PureLink^™^ Micro-to-Midi Total RNA Purification System (Invitrogen, UK), according to the manufacturer’s instructions. Briefly, 16 h after treatment, cells were trypsinized, washed in MEM with FBS for trypsin inactivation and resuspended in PBS. After centrifugation, all excess liquid was removed and 1 mL of TRI REAGENT^™^ was added to each sample. Samples were mixed and centrifuged. The aqueous phase was transferred to a fresh microcentrifuge tube and an equal amount of 70% ethanol was added. Samples were transferred to a PureLink^™^ Micro-to-Midi Total RNA Purification System column (Invitrogen, UK) and processed according to the manufacturer’s protocol. All samples were washed from the column with 75 μl of RNAse free water.

The quality of isolated RNA was checked on a Bioanalyzer 2100 (Agilent, USA) using RNA 6000 Nano Labchip (Agilent) and 6000 RNA ladder as reference (Ambion). Concentration and quantity of RNA was determined with ND-1000 (Nanodrop, USA).

Preparation of aaRNA was performed with an Amino Allyl MessageAmp^™^ II aRNA Amplification Kit (Ambion) according to the manufacturer’s recommendations. For each hybridization, we labelled 5 μg of control (Cy3) and 5 μg of treated (Cy5) mRNA. After removing the excess dye, the RNAs were dissolved in Nexterion Hybridization solution (Schott Nexterion). Since single-strand oligonucleotides are strong electrolytes care was taken to keep the ionic strength of the medium and temperature the same in all experiments.

Microarrays were prepared with Human Apoptosis Subset v2.0 and Human Cancer Subset v3.0 (Operon, Germany) 70mer oligonucleotides and Nexterion 70mer Oligo Microarraying Kit (Schott Nexterion, Germany) slides. Single array contained 2698 different genes, each gene being replicated at least 4 times on each array. Oligonucleotides were spotted using an MG1000 spotter (MicroGrid, USA), immobilised and stored according to the manufacturer’s instructions (Schott Nexterion). All hybridisations were performed on HS400 (Tecan, Austria) according to the manufacturer’s instructions (Schott Nexterion). We used an LS200 scanner (Tecan) at 6 μm resolution for scanning the microarrays.

Data was analysed using Array-Pro Analyzer 4.5 (Media Cybernetics, Bethesda, MD, USA) for feature extraction after imaging of microarrays. Acuity 4.0 (Molecular devices, USA) was used for filtration of bad signals, LOWESS normalization, and microarray data analysis. Features showing signal intensity of more than 65000 were flagged as bad. Features with signal less than 2 times the intensity of background or coefficient of variation (CV, ratio between standard deviation of the background and the median feature intensity) greater than 0.3 were considered not significantly expressed and were filtered out. Log_2_ ratios were normalized using LOWESS fit.^30^ Median from four replicates was used to calculate average gene expression for single sample. Differentially expressed genes were selected based on direct comparison between treated and untreated cells where difference in expression was used as cut off for detection. Genes showing differential expression of more than 1.5-fold in all replicates were considered as differentially expressed. The standard error was calculated for all differentially expressed genes.

Gene enrichment analysis was performed using WebGestalt program.^31^ Sets of differentially expressed genes were compared to the original gene dataset. The hypergeometric test was used for GO category enrichment evaluation. The method by Benjamini and Hochberg was used for multiple test adjustment. Significance level was set to either 0.05 or Top10 (to identify the 10 categories with the most significant p values). A cut-off for the minimum number of genes required to test per category was 2.

### Validation of microarrays by Quantitative Real-Time PCR (qRT-PCR) Analysis

Samples of total RNA isolated for preparation of microarray assay were used also for validation of microarray results by qRT-PCR analysis. One hundred ng of isolated total RNA was reverse transcribed using Quanti-Tect Reverse Transcription Kit (Qiagen, Germany). In the first step, genomic DNA in the samples was eliminated by addition of gDNA Wipeout Buffer and incubation at 42°C for 2 min. A master mix for reverse transcription prepared from Quantiscript Reverse Transcriptase, Quantiscript RT Buffer, and RT Primer Mix, was added to the samples and incubated at 42°C for 15 minutes. The samples were further incubated at 95°C for 3 min to inactivate Quantiscript Reverse Transcriptase. cDNA samples were either used directly in qRT-PCR or stored at −20°C for long term storage. Similarly, 1 μg of total RNA from the control sample was reverse transcribed and serially diluted to prepare standard curve for quantification of the qRT-PCR results.

Five genes were selected for validation by qRT-PCR based on their involvement in metastatic process (*LASS2*, *PHLDA2*, *PRKCD*, *VIM*, and *LAMB3*) and one gene was selected based on its expression pattern after exposure to electric pulses used for ECT (*HSPA1B*). Specific Taqman Gene Expression Assays (Applied Biosystems, USA) were used to measure expression of *LASS2*, *PHLDA2*, *PRKCD*, *VIM*, *LAMB3,* and *HSPA1B* genes in control and treated samples (Table 1). Human *GAPDH* (Pre-Developed TaqMan Assay Reagents, Applied Biosystems) was used as an endogenous control. qRT-PCR reactions were performed using a TaqMan Universal PCR Master Mix II (Applied Biosystems) and 7300 Real Time PCR System (Applied Biosystems). Thermal cycling conditions included initial UNG incubation at 50°C for 2 min, followed by polymerase activation at 95°C for 10 min and 50 cycles of 15 seconds at 95°C and 1 min at 60°C. Data was analysed using Applied Biosystems SDS v1.3.1. A standard curve method was used for quantitative analysis. The expression level of selected genes was normalized to the expression of *GAPDH* in each sample and is presented as a fold-change in expression compared to control samples.

## Results

### Cell survival after electrochemotherapy

**FIGURE 1:**
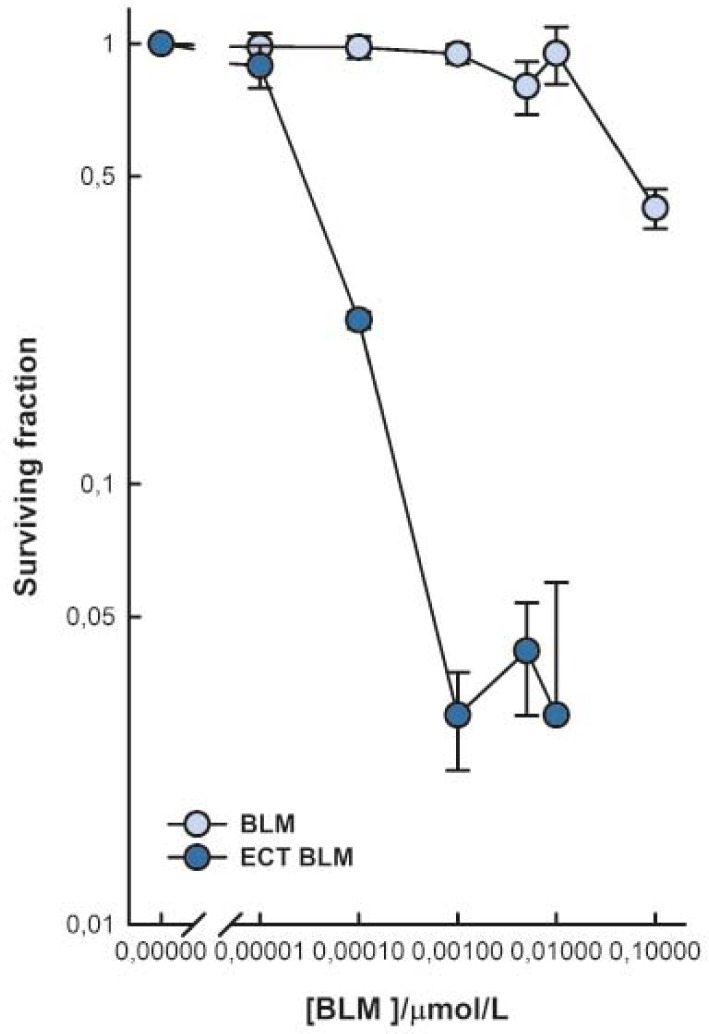
Cell survival after exposure to BLM and electrochemotherapy with BLM. The surviving fraction of cells exposed to electrochemotherapy was normalized to electric pulses treatment alone. Data are expressed as mean value ± standard error of the mean.

Cell survival after ECT with BLM was determined by a clonogenic assay. Throughout the range of tested BLM concentrations, melanoma SK-MEL28 cells exposed to electroporation were more sensitive to BLM than the unexposed cells (Figure 1). BLM treatment significantly reduced cell survival at 0.1 μM BLM in comparison to untreated cells, whereas ECT with BLM significantly reduced cell survival already at 0.01 nM BLM. Exposure of cells to electroporation resulted in increased BLM cytotoxicity as determined at IC_50_ value (0.03 μM for BLM treatment and 0.038 nM for ECT with BLM). Overall, electroporation increased BLM cytotoxicity 800-fold.

**FIGURE 2:**
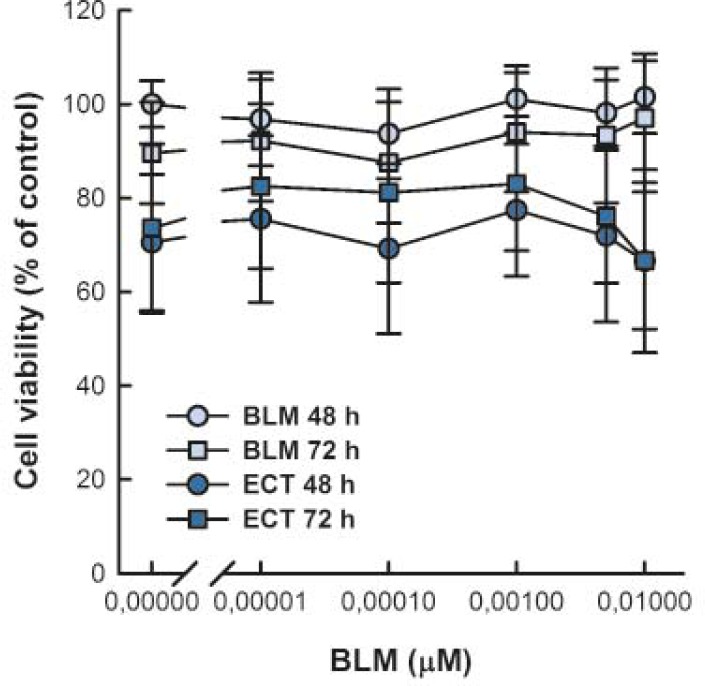
Viability of SK-MEL28 cells 48 h and 72 h after BLM treatment or electrochemotherapy with BLM. Cell viability was normalized to control cells at 48 h. Data are expressed as mean value ± standard error of the mean.

Viability of SK-MEL28 cells was determined by MTS assay 48 and 72 h after treatment to assure that the changes in migration and invasion of SK-MEL28 cells are not due to cell death (Figure 2). Cell viability was not affected by different BLM concentrations at 48 and 72 h post-treatment in comparison to control cells at 48 h. Similarly, ECT with BLM did not affect cell viability at the time of determination of metastatic potential.

### Migration, invasion and adhesion assays

**FIGURE 3:**
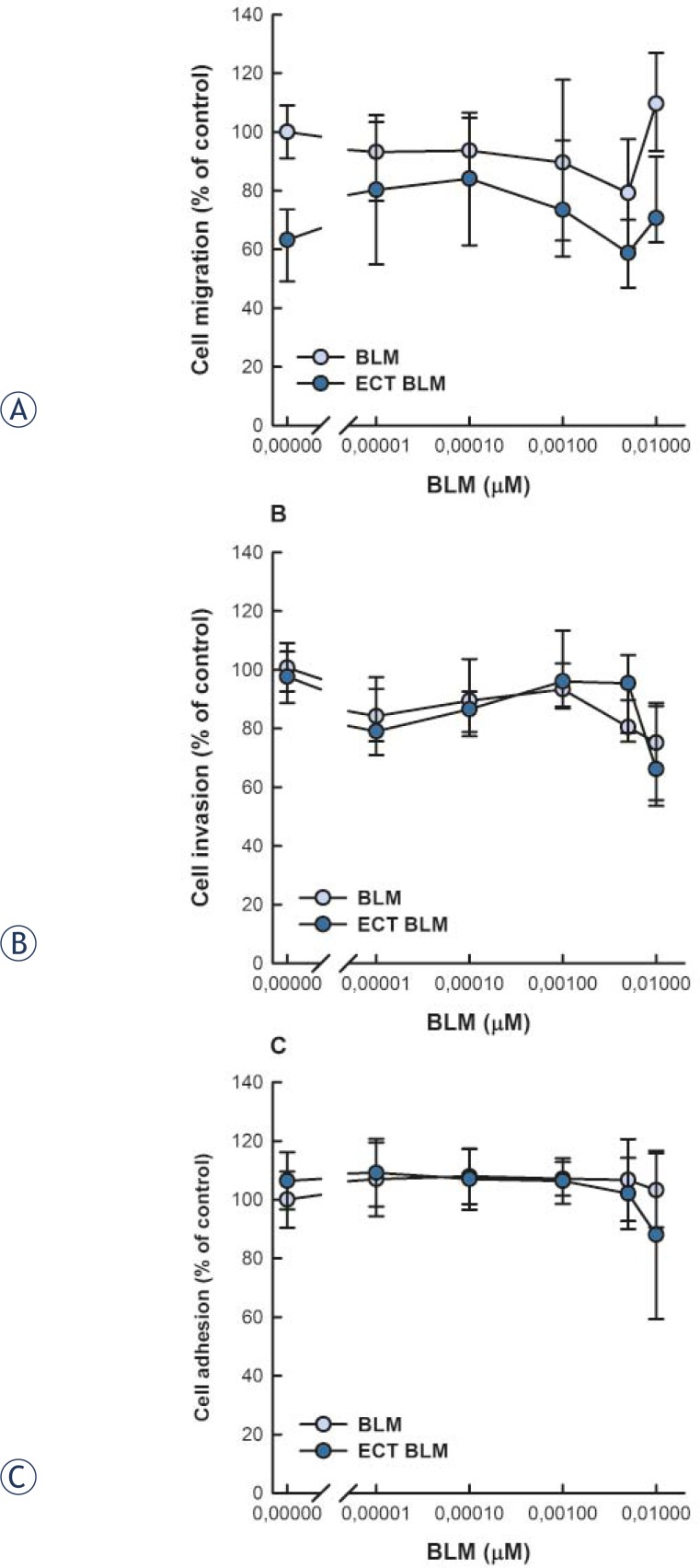
Migration (A), invasion (B) and adhesion (C) of human melanoma SK-MEL28 cells 72 h after BLM treatment or electrochemotherapy with BLM. Data are expressed as mean value ± standard error of the mean.

SK-MEL28 cells that were viable 48 h post-treatment were plated in cell inserts for migration and invasion assay and incubated another 24 h before the number of viable cells in each compartment was determined by MTT assay. The experimental system allowed for about 23.5% of the control cells to migrate and about 17.3% of the control cells to invade through the Matrigel. Cell migration and invasion were not affected either by BLM treatment or ECT with BLM throughout the tested concentrations (Figure 3A and 3B). Interestingly, cell migration was lower after ECT in comparison to control and BLM treated cells, but this was not statistically significant.

Furthermore, adhesion of SK-MEL28 cells to Matrigel was determined 48 hours post-treatment. Throughout the tested concentrations, neither BLM alone nor ECT with BLM affected cell adhesion in comparison to control cells (Figure 3C).

### Microarray assay

The difference in gene expression was identified by comparison of malignant melanoma cells exposed to either BLM alone, electric pulses alone, or ECT with BLM to control cells. A total of 2698 genes involved in cancer development were analysed. Exposure of cells to BLM alone yielded 14 differentially expressed genes (0.5% of the investigated genes) (Table 2). Exposure of cells to electric pulses yielded 34 differentially expressed genes (1.3% of the investigated genes) (Table 3). Similarly to exposure to electric pulses, ECT with BLM yielded 34 differentially expressed genes (1.3% of the investigated genes) (Table 4). There were no common genes differentially expressed in all three groups. However, the expression of 2 genes, *CYP2A7* and *SGK*, was down-regulated after exposure to BLM and ECT with BLM, whereas the expression of 5 genes was differentially expressed after exposure to electric pulses and ECT with BLM, of which *TNFRSF14*, *TBCA* and *RPA3* genes were down-regulated, and *AD7c-NTP* and *HSPA1B* up-regulated.

Further, we analysed sets of differentially expressed genes using the Gene Ontology Tree Machine program to identify biological processes involved in response to exposure to BLM or electroporation. Differentially expressed genes (Table 2, 3, 4) were compared to the list of all genes included on the microarray to identify any significant gene enrichment in comparison to the original gene dataset. We found gene enrichment in differentially expressed genes involved in translation, ribosomal small subunit biogenesis and negative regulation of collagen metabolic process in cells exposed to electric pulses only. There was no gene enrichment after exposure to BLM alone, or ECT. In addition, there was no significant gene enrichment in biological processes related to metastatic potential after exposure to BLM or ECT with BLM.

### Validation of microarrays by Quantitative Real-Time PCR Analysis

**FIGURE 4:**
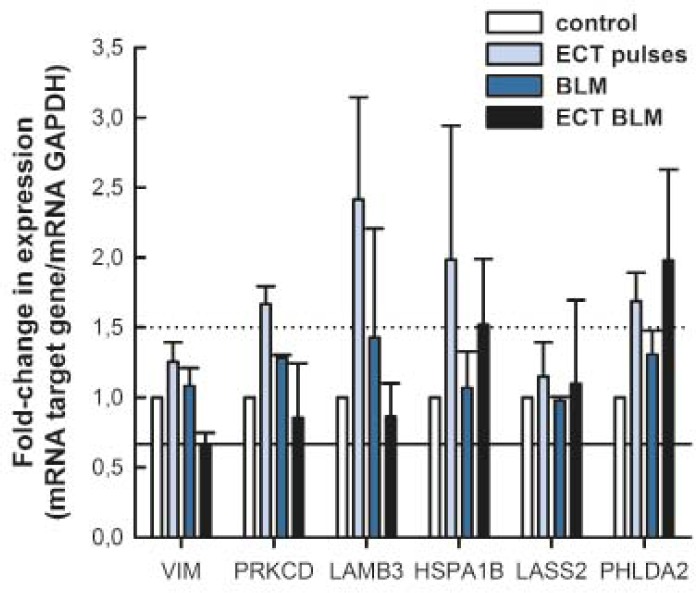
Fold-change in expression of selected genes by qRT-PCR. Dotted line denotes a 1.5-fold up-regulation and solid line a 1.5-fold down-regulation of gene expression. Data are expressed as mean value ± standard error of the mean.

Five genes that were differentially expressed in microarrays were selected for validation by qRT-PCR based on their involvement in metastatic process (*LASS2*, *PHLDA2*, *PRKCD*, *VIM*, and *LAMB3*) and one gene was selected based on its expression pattern after exposure to electric pulses used for ECT (*HSPA1B*). Expression levels of selected genes were analysed in all sample groups regardless of their expression level measured by microarrays. The expression level of selected genes was determined as a fold-change in expression compared to control samples. None of the tested genes was more than 1.5-fold down-regulated, whereas several genes were up-regulated more than 1.5-fold after treatment (Figure 4). *PRKCD* and *LAMB3* were up-regulated more than 1.5-fold after exposure to electric pulses alone. *HSPA1B* was up-regulated more than 1.5-fold after exposure to electric pulses alone or ECT with BLM. *PHLDA2* was up-regulated more than 1.5-fold after exposure to electric pulses alone and ECT with BLM. The expression levels of *VIM* and *LASS2* were not differentially expressed after treatment as detected by qRT-PCR. Our results demonstrated that the expression levels of selected genes evaluated by microarrays or qRT-PCR were similar and did not differ significantly (Table 5). It is of interest that 3 genes (*LAMB3*, *PRKCD* and *PHLDA2*) were found to be more than 1.5-fold up-regulated by qRT-PCR after exposure to electric pulses, but not by microarrays (Table 5).

## Discussion

The aim of this study was to evaluate for the first time the effect of ECT with BLM on metastatic potential of melanoma cells SK-MEL28 *in vitro*. Our results demonstrated that ECT with BLM does not affect metastatic potential of melanoma cells *in vitro*. In addition, a low number of genes were differentially expressed after ECT with BLM. Gene expression of *LASS2*, *PHLDA2*, *PRKCD*, *VIM*, *and LAMB3* involved in metastatic processes was minimally up-regulated, which was confirmed by qRT-PCR. Furthermore, *HSPA1B* that was up-regulated in response to electric pulses was also minimally up-regulated. However, this up-regulation did not result in biological response. All together these data indicate that metastatic potential of melanoma cells is not increased by ECT with a wide range of BLM concentrations.

Cytotoxicity potentiation after exposure to electric pulses is due to increased drug accumulation.^32^ Cytotoxicity potentiation was demonstrated in many *in vitr*o studies on different tumour cell lines. In comparison to other cell lines, melanoma cells SK-MEL28 were the most sensitive cells to treatment with BLM alone.^33^–^36^ However, BLM cytotoxicity was considerably potentiated by 800 fold after exposure of cells to electric pulses and is in the middle range of potentiation factors for different cell lines.

In the last decade, many studies focused their attention to a controversial problem of therapy induced metastases. The effects of surgery on tumour growth were proposed in the late 50’s and were later confirmed by experimental studies (reviewed in 37). To improve surgical resection of tumours different fluorescence imaging agents can be used to detect and remove any residual tumour cells.^38^ It is important to remove all tumour cells because surgical resection of a tumour changes the microenvironment of the wound site and provides a tumour growth favourable microenvironment.^4^–^6^ Similar findings for therapy induced metastases were demonstrated also for irradiation therapy. It can promote malignant behaviour of various cancer cell types *in vitro*^8^–^11^ and *in vivo.*^7^,^9^,^10^,^39^ Changes in cell migration and invasion are dependent both on the type and dose of radiation used.^7^–^11^ For melanomas, increased probability for developing metastatic disease after subcurative irradiation therapy was demonstrated.^7^ This can be explained by melanomas having a high intrinsic capacity for repair of sublethal DNA damage caused by irradiation.^40^

Contrary to the studies on surgery and radiation induced metastases, not much is known about the effect of ECT, which is also a local treatment, on metastatic potential of tumour cells. In the clinical studies, there were no reports of increased metastatic spread of tumours after ECT.^18^,^20^,^41^ Relevant clinical problem are the cells surviving ECT due to insufficient drug distribution or suboptimal electroporation of the tissue.^21^,^22^,^42^ Therefore, it is important to evaluate the effect of ECT on tumour cells surviving ECT.

We chose a melanoma cell line SK-MEL28 as a study model because these cells were characterized having a high migratory potential.^28^ To simulate clinical conditions, SK-MEL28 cells were exposed to either BLM treatment or ECT with BLM and after 48 h viable cells were plated for cell migration, invasion and adhesion assays. We used cell culture inserts with porous membrane that are widely used in *in vitro* studies of cell migration and invasion because metastatic potential is directly linked to tumour cell migration and invasion capability.^8^,^28^ Basement membrane is the critical barrier to the invasion of the tumour cells.^28^ For cell invasion assay, the inserts were coated with a layer of Matrigel, a mixture of basement membrane proteins, routinely used in the studies of cell invasion to mimic extracellular matrix barrier.^27^,^28^ Our experimental conditions allowed for about 23.5% of the control cells to migrate and about 17.3% of the control cells to invade through the Matrigel. A range of BLM concentrations from 0.01 nM to 0.01 μM were evaluated either alone or in combination with electroporation. As expected, we demonstrated that neither cell migration nor cell invasion was affected after electrochemotherapy with different BLM concentrations. Similarly, no effect of BLM treatment alone or of exposure to electric pulses on cell migration and invasion was seen. Our results also show that smaller proportion of control cells was able to invade through the Matrigel layer. This is expected because other factors, such as proteolytic enzymes, are involved in cell invasion processes.

Changes in migration and invasion activities can be affected by the differing ability of cells to adhere to membranes.^8^,^28^ In this regard, cell adhesion to Matrigel was tested on cells that were viable 48 hours after treatment and we demonstrated that there was no change in cell adhesion after BLM treatment or electrochemotherapy with BLM. Because the migratory and invasive cells were determined by colorimetric MTT assay that is directly proportional to the number of viable cells the results of the migration and invasion assay can be affected by cell death after therapy. To avoid this problem, the number of cells was adjusted prior to seeding cells in the cell culture inserts for migration and invasion assay. In addition, we also demonstrated that there was no difference in cell proliferation rate of the treated cells in the 24-hour interval of migration and invasion assays.

The results of this study complement our previous study where we demonstrated that cell migration and invasion of SK-MEL28 cells is not affected by suboptimal exposure to ECT with cisplatin.^25^ Furthermore, the results of both studies are supported by the findings of various clinical studies where it was demonstrated that none of the lesions in complete response after ECT with BLM or cisplatin relapsed during the follow-up of 21 months.^18^,^20^,^41^

However, an important aspect of therapy induced metastases is also the modulation of tumour microenvironment in a way to promote metastatic behaviour. Different mechanisms are involved in induction and promotion of metastasis. Also, different treatments affect metastatic potential in different ways as well as through different mechanisms. In many studies up-regulation of β_3_ and β_1_ integrins was observed and correlated with increased cell migration and invasion after exposure to different types of radiation.^8^–^10^,^43^ Furthermore, up-regulation of matrix metalloproteinase-9 (MMP-9), metalloproteinase-2 (MMP-2), BCL-2, BCL-X_L_, integrin-linked kinase (ILK), and interleukin-9 was observed in different tumour cells in response to different types of radiation.^10^,^11^,^39^,^43^ It was also observed that tumours regrowing after radiation treatment can have increased metastatic potential because of radiation-induced hypoxia and hypoxia-induced up-regulation of gene products promoting metastasis.^7^

To evaluate the effect of ECT with BLM on gene expression, we prepared microarrays with 2698 genes involved in the development of cancer. The number of differentially expressed genes after exposure to BLM or ECT with BLM was very low (ranging from 0.5% to 1.3% of all genes on the microarray). Also, fold-expression of differentially expressed genes was low. Only 7 of differentially expressed genes after exposure to BLM, electric pulses or ECT with BLM, of which 6 were down-regulated, had fold-expression above 2-fold after ECT with BLM or exposure to electric pulses. These differentially expressed genes are involved in various biological processes.

To identify possible biological processes involved in the response to BLM or ECT with BLM, we used the WebGestalt program for gene enrichment analysis. The original dataset of genes was compared to differentially expressed genes after BLM treatment or ECT with BLM to identify any significant gene enrichment. There was no gene enrichment among the differentially expressed genes after BLM treatment or ECT with BLM. Similar results were demonstrated for electric pulses used for ECT or electrogene therapy where significant gene enrichment was observed, however not in categories related to metastasis formation.^24^ These results can be correlated to studies carried out on mouse muscles, showing that electroporation does not induce significant changes in gene expression. DNA electrotransfer to mouse muscle induces only small changes in the expression of cytoskeletal and intracellular transport proteins, while no significant changes in gene expression profiles of proteins involved in stress, cell death and inflammation or muscle regeneration were observed in response to EP delivery.^44^,^45^ In our *in vitro* study, there was no change in expression of genes involved in inflammation as only tumour cells were included for gene expression analysis. On the other hand, *in vivo* study of mouse melanoma demonstrated that plasmid DNA encoding reporter luciferase gene alone or in combination with electric pulses and different electroporation protocols affects endogenous gene expression.^46^ Increased levels of mRNA and protein levels for inflammatory chemokines and cytokines were observed 4 h after gene electrotransfer and by 24 h, the expression levels of mRNAs and proteins were already considerably reduced.^46^

A commonly used validation tool for confirming gene expression results from microarray analysis is qRT-PCR.^47^,^48^ From differentially expressed genes after BLM treatment and ECT, we selected 6 genes and we evaluated their mRNA expression levels by qRT-PCR. The direction of change in gene expression was the same by both microarrays and qRT-PCR for 5 genes (*HSPA1B*, *LASS2*, *PHLDA2*, *PRKCD*, and *VIM*) with differential expression by microarrays. Interestingly, 3 genes (*LAMB3*, *PRKCD* and *PHLDA2*) were found to be more than 1.5-fold up-regulated by qRT-PCR after exposure to electric pulses, but not by microarrays. However, this changes in gene expression can be considered minor as only one (*LAMB3*) of the genes was more than 2-fold up-regulated. It was demonstrated before that several parameters can affect correlation of data between microarray and qRT-PCR results, one of them being fold change in gene expression.^48^ Decreased correlations for genes with minor changes in expression (less than 1.5) using probe based qRT-PCR and oligonucleotide microarrays were reported.^47^,^48^ Also, minor changes in gene expression seen by different methods can be a consequence of different chemistries used in the assays or differences in specific sequence targets.

*PRKCD* (Protein kinase C, delta type), *VIM* (Vimentin), and *LAMB3* (Laminin β3 chain precursor) are known to be involved in the metastatic process. Increased expression of *PRKCD* increases metastatic potential of melanoma cells.^49^–^51^ However, other molecules are also involved in the *PRKCD*-induced increased cell invasiveness in melanoma cells.^49^ Up-regulation of *VIM* is typical for aggressive cell lines with high metastatic potential, whereas down-regulation of *VIM* decreases migration and invasion of breast and colon carcinoma.^49^,^52^–^54^ *LAMB3* codes β_3_ subunit of laminin-5, a protein involved in migration, invasion and metastasis formation in tumour cells.^55^–^57^ Down-regulation of β_3_ subunit in tumour cells is associated with the loss of basal membrane in invasive carcinomas.^55^ We speculate that changes in *PRKCD*, *VIM* and *LAMB3* expression were not sufficient to increase metastatic behaviour of melanoma cells SK-MEL28 or that deregulated expression of other genes must be present to increase metastatic behaviour. Up-regulation of *PHLDA2* is associated with Fas-receptor mediated apoptosis^58^ and increased expression of *LASS2* leads to increased cell death.^59^ Up-regulation of both *PHLDA2* and *LASS2* after ECT with BLM is likely associated with apoptosis induction in cells exposed to cytotoxic concentrations of BLM. It is known that BLM induces apoptosis.^60^ *HSPA1B* was the only gene with a specific pattern of gene expression as it was up-regulated after exposure to electroporation and ECT with BLM. *HSPA1B* up-regulation was observed also after electroporation with electric pulses used in ECT and electrogene therapy protocols^24^ as well as after ECT with cisplatin.^25^ Increased expression of *HSPA1B* can be a consequence of changes in cell membrane structure after electroporation because changes in cell membrane structure can initiate signalling pathways to increase expression of heat shock proteins.^61^,^62^

## Conclusions

To conclude, in this study we evaluated metastatic potential of melanoma cells SK-MEL28 that survived ECT with BLM at the *in vitro* level by assessing cell migration, invasion and adhesion. We demonstrated no change in these cell properties, and hence no change in metastatic potential after ECT with BLM. Furthermore, a low number of tumourigenesis related genes were differentially expressed and there was no gene enrichment in metastasis promoting genes. Together with our previous findings on ECT with cisplatin, we can confirm that ECT is a safe local treatment modality of superficial tumours that does not alter tumorigenic and metastatic properties of tumour cells that survived ECT with BLM.

## Figures and Tables

**TABLE 1:** Taqman Gene Expression Assays for selected genes

**Gene Symbol**	**Gene Name**	**Assay ID**	**Probe Sequence**
*LASS2*	LAG1 homolog, ceramide synthase 2	Hs00604577_m1	CTGCCGCCGGGATGCTCCAGACCTT
*PHLDA2*	Pleckstrin homology-like domain, family A, member 2	Hs00169368_m1	CCCGCCGCGGGCCATACGCTGGACG
*PRKCD*	Protein kinase C, delta	Hs00178914_m1	AGGCCCAAAGTGAAGTCACCCAGAG
*VIM*	Vimentin	Hs00185584_m1	CCGGGAGAAATTGCAGGAGGAGATG
*LAMB3*	Laminin, β3	Hs00165078_m1	CACCCAGTATGGCGAGTGGCAGATG
*HSPA1B*	Heat shock 70 kDa protein 1B	Hs01040501_sH	CGCGGATCCCGTCCGCCGTTTCCAG

**TABLE 2:** Differentially expressed genes after treatment with bleomycin

**DOWN-REGULATED GENES**		

Gene symbol (RefSeq^a^)	Fold expression^b^	Protein product
*CYP2A7* (NM_000764)	1.9 ± 0.3	Cytochrome p450 2A7
*DRPLA* (NM_001940)	1.8 ± 0.2	Atrophin1
*CGB5* (NM_033142)	1.7 ± 0.004	Choriogonadotropin beta chain precursor
*RPL31* (NM_000993)	1.7 ± 0.1	Ribosomal protein L31
*SGK* (NM_005627)	1.7 ± 0.2	Serine/threonineprotein kinase SGK
*MYL12* (NM_006471)	1.6 ± 0.03	Myosin regulatory light chain 2
*EEF1A1* (NM_001402)	1.5 ± 0.07	Elongation factor 1alpha 1
*CGB5* (NM_033142)	1.5 ± 0.05	Choriogonadotropin beta chain precursor

**UP-REGULATED GENES**		

Gene symbol (RefSeq^a^)	Fold expression^b^	Protein product
*ARHD* (NM_014578)	1.8 ± 0.3	Rho-related GTP-binding protein rhod
*INSM1 (NM_002196)*	1.6 ± 0.07	Zinc finger protein Ia1
*RBL2* (NM_005611)	1.6 ± 0.1	Retinoblastoma-like protein 2
*HOXA4* (NM_002141)	1.6 ± 0.1	Homeobox protein HOXA4
***PRKCD*** (NM_006254)	1.5 ± 0.04	Protein kinase C, delta type
*GNRHR* (NM_000406)	1.5 ± 0.03	Gonadotropin-releasing hormone receptor

a Gene accession number from NCBI Reference Sequence database.

b Values represent mean fold-expression (calculated from the log_2_ ratio) and standard error of pooled data from 3 independent experiments. Gene expression of genes in **bold** was validated by qRT-PCR method.

**TABLE 3:** Differentially expressed genes cells after application of electric pulses

**DOWN-REGULATED GENES**		

*Gene symbol* (RefSeq^a^)	Fold expression^b^	Protein product
*RPL31* (NM_000993)	3.0 ± 0.8	ribosomal protein L31
*RPS17* (NM_001021)	2.3 ± 0.9	40S ribosomal protein S17
*TBCA* (NM_004607)	2.2 ± 0.5	tubuline specific chaperone A
*PPIA* (NM_021130)	2.0 ± 0.5	peptidylprolyl cistrans isomerase A
*S100B* (NM_006272)	2.0 ± 0.5	S100 protein
*MYL9* (NM_006471)	1.9 ± 0.3	myosin regulatory light chain 2
*RPA3* (NM_002947)	1.9 ± 0.5	replication protein A
*NQO1* (NM_000903)	1.9 ± 0.3	NAD(P)H dehydrogenase 1
*RPS6* (NM_001010)	1.8 ± 0.1	40S ribosomal protein S6
*H4FN* (NM_175054)	1.7 ± 0.1	histone H4
*ITGB4* (NM_000213)	1.7 ± 0.2	integrin beta 4 precursor
*EEF1A1* (NM_001402)	1.7 ± 0.1	elongation factor-1 α1
*CD28* (NM_006139)	1.7 ± 0.1	CD28 antigen precursor
*H3F3A* (NM_002107)	1.7 ± 0.2	histone H3.3
*CASP9* (NM_001229)	1.7 ± 0.1	caspase 9 precursor
*TNFRSF14* (NM_003820)	1.6 ± 0.1	TNF receptor superfamily member 14 precursor
*CGB5* (NM_033142)	1.6 ± 0.1	choriogonadotropin beta chain precursor
*RPH3AL* (NM_006987)	1.5 ± 0.06	rabphilin 3A like
*TFDP1* (NM_007111)	1.5 ± 0.07	transcription factor Dp-1
*CST3*(NM_000099)	1.5 ± 0.06	cystatin C precursor

**UP-REGULATED GENES**		

*Gene symbol* (RefSeq^a^)	Fold expression^b^	Protein product
*IL6* (NM_000600)	2.0 ± 0.5	interleukin 6 precursor
***HSPA1B*** (NM_005346)	1.9 ± 0.1	heat shock 70 kDa protein 1
*RBL2* (NM_005611)	1.7 ± 0.2	retinoblastoma like protein 2
*CCNF* (NM_001761)	1.7 ± 0.1	G2/mitotic specific cyclin F
*CRABP2* (NM_001878)	1.7 ± 0.1	retinoic acid binding protein II
*GLIPR1* (NM_006851)	1.7 ± 0.2	glioma pathogenesis related protein
*AD7c-NTP* (NM_014486)	1.7 ± 0.2	neuronal thread protein
*CDC25C* (NM_001790)	1.6 ± 0.03	M phase inducer phosphatase 3
*RBBP4* (NM_005610)	1.6 ± 0.1	chromatin assembly factor 1 subunit C
*HOXA4* (NM_002141)	1.6 ± 0.1	homeobox protein HOXA4
*MATR3* (NM_018834)	1.5 ± 0.02	matrin 3
*DNAJB1* (NM_006145)	1.5 ± 0.05	DNAJ homolog subfamily B member 1
*RIN2* (NM_018993)	1.5 ± 0.04	Ras and Rab interactor 2
*RB1* (NM_000321)	1.5 ± 0.02	retinoblastoma 1

a Gene accession number from NCBI Reference Sequence database.

b Values represent mean fold-expression (calculated from the log_2_ ratio) and standard error of pooled data from 3 independent experiments. Gene expression of genes in **bold** was validated by qRT-PCR method.

**TABLE 4:** Differentially expressed genes after electrochemotherapy with bleomycin

**DOWN-REGULATED GENES**		

*Gene symbol* (RefSeq^a^)	Fold expression^b^	Protein product
*SGK* (NM_005627)	2.8 ± 0.07	Serine/threonine protein kinase
*TNFRSF14* (NM_003820)	1.9 ± 0.2	Tumour necrosis factor receptor superfamily member 14 precursor
*SPARC* (NM_003118)	1.9 ± 0.3	SPARC precursor
*TBCA* (NM_004607)	1.9 ± 0.3	Tubulinspecific chaperone A
*LDHA* (NM_005566)	1.7 ± 0.2	L-lactate dehydrogenase A chain
*RPA3* (NM_002947)	1.6 ± 0.1	Replication protein A 14 kDa subunit
*NT5E* (NM_002526)	1.6 ± 0.03	5’nucleotidase precursor
*HSPE1* (NM_002157)	1.6 ± 0.09	Heat shock 10kDA protein 1
*TUBA1* (NM_006082)	1.6 ± 0.02	Tubulin alpha1 chain
*TOP2A* (NM_001067)	1.6 ± 0.2	Topoisomerase (DNA) II alpha
*CYP2A7* (NM_000764)	1.5 ± 0.2	Cytochrome p450 2A7
*CALM3* (NM_001743)	1.5 ± 0.08	Calmodulin
*C1QBP* (NM_001212)	1.5 ± 0.09	Complement component 1, q subcomponent binding protein
***VIM*** (NM_003380)	1.5 ± 0.1	Vimentin
*PSMB7* (NM_002799)	1.5 ± 0.2	Proteasome subunit beta type 7 precursor
*NME1* (NM_000269)	1.5 ± 0.1	Nucleoside diphosphate kinase A
*RET* (NM_020975)	1.5 ± 0.08	Protooncogene tyrosineprotein kinase receptor ret precursor
*YWHAZ* (NM_003406)	1.4 ± 0.02	Protein kinase C inhibitor protein1
*PDCD5* (NM_004708)	1.4 ± 0.08	Programmed cell death protein 5
*ID3* (NM_002167)	1.4 ± 0.08	DNA binding protein inhibitor
*PRSS11* (NM_002775)	1.4 ± 0.2	Serine protease HTRA1 precursor
*MERTK* (NM_006343)	1.4 ± 0.1	Protooncogene tyrosineprotein kinase MER precursor

**UP-REGULATED GENES**		

*Gene symbol* (RefSeq^a^)	Fold expression^b^	Protein product
*TAX1* (NM_014604)	1.7 ± 0.3	TAX interaction protein 1
*TNA* (NM_003278)	1.7 ± 0. 4	Tetranectin precursor
*AD7c-NTP* (NM_014486)	1.7 ± 0.2	Neuronal thread protein
*CTSK* (NM_000396)	1.6 ± 0.2	Cathepsin K precursor
***HSPA1B*** (NM_005346)	1.6 ± 0.2	Heat shock 70 kDa protein 1
***PHLDA2*** (NM_003311)	1.6 ± 0.1	Tumour suppressing subtransferable candidate 3
*TTYH1* (NM_020659)	1.5 ± 0.3	Tweety homolog 1
*JUND* (NM_005354)	1.5 ± 0. 1	Jun D proto-oncogene
***LASS2*** (NM_013384)	1.5 ± 0.2	Tumour metastasis suppressor
*BTF3* (NM_001207)	1.4 ± 0.1	Transcription factor BTF3
*ARG2* (NM_001172)	1.4 ± 0.1	Arginase II
*CBS* (NM_000071)	1.4 ± 0.1	Cystathionine betasynthase
*RPLP0* (NM_053275)	1.4 ± 0.1	60s acidic ribosomal protein P0

a Gene accession number from NCBI Reference Sequence database.

b Values represent mean fold-expression (calculated from the log_2_ ratio) and standard error of pooled data from 3 independent experiments. Gene expression of genes in **bold** was validated by qRT-PCR method.

**TABLE 5:** Comparison of differential gene expression by microarrays and qRT-PCR for selected genes after bleomycin treatment, electroporation or electrochemotherapy with bleomycin

**gene**	**fold-change ± st. dev.**		**treatment**
	microarrays	qRT-PCR	
**up-regulated genes^a^**			
**HSPA1B**	1,9 ± 0,1	2,0 ± 1,0	EP
	1,6 ± 0,2	1,5 ± 0,5	ECT BLM
**LASS2**	1,5 ± 0,2	1,1 ± 0,6	ECT BLM
**PHLDA2**	1,6 ± 0,1	2,0 ± 0,7	ECT BLM
**PRKCD**	1,5 ± 0,04	0,9 ± 0,4	BLM

**down-regulated genes^a^**			

**VIM**	1,5 ± 0,1	1,5 ± 0,2	ECT BLM

**no change in expression^a^**			

**LAMB3**	1^b^	2,4 ± 0,7	EP
**PRKCD**	1^b^	1,7 ± 0,1	EP
**PHLDA2**	1^b^	1,7 ± 0,2	EP

a indicates change in expression as determined by microarrays.

b indicates there was no change in gene expression determined by microarray analysis.
